# It takes a community: a landscape analysis of global health research consortia

**DOI:** 10.1136/bmjgh-2019-001450

**Published:** 2019-08-16

**Authors:** Amelia VanderZanden, Etienne V Langlois, Abdul Ghaffar, Asaf Bitton, Jocelyn Fifield, Lisa R Hirschhorn

**Affiliations:** 1 Ariadne Labs, Brigham and Women's Hospital & Harvard T.H. Chan School of Public Health, Boston, Massachusetts, USA; 2 Alliance for Health Policy and Systems Research, WHO, Geneva, Switzerland; 3 Division of General Medicine, Brigham and Women's Hospital, Boston, Massachusetts, USA; 4 Northwestern University Feinberg School of Medicine, Chicago, Illinois, USA

**Keywords:** research consortium, research network, primary health care, landscape analysis

## Abstract

**Background:**

The increased recognition of the core role of effective primary healthcare has identified large gaps in the knowledge of components of high-quality primary healthcare systems and the need for resources positioned to better understand them. Research consortia are an effective approach to generate evidence needed to address knowledge and evidence gaps and accelerate change. However, the optimal design of consortia and guidance on design decisions is not well studied. We report on a landscape analysis to understand global health research consortium models and major design decisions that inform model choice.

**Methods:**

We conducted a landscape analysis to identify health-related research consortia typologies and explore decision processes leading to their design and implementation. We identified and reviewed 195 research consortia, extracted data on organisation, characteristics and operations for 115 and conducted 14 key informant interviews representing 13 consortia. We analysed interviews using thematic content analysis using results to develop categories of major design choices and research consortia models, structures and processes.

**Results:**

Across a wide range of research consortia, the structure and function were determined by nine key design decisions that were mapped to three domains: scope: including mission and area of focus; organisational structure: including role and location of the core entity, choice of leader, governance and membership eligibility and responsibility; and funding decisions: including the funding source for research consortia operations and the funding sources and process for consortium research.

**Discussion:**

Research consortia showed important heterogeneity across the nine decision points studied and based on their goals, needs and resources. These decisions and the three emerging domains (scope, organisation and funding) offer a potential framework for new research consortia and inform the design of a proposed primary health care research consortium intended to accelerate research to improve primary health care in LMICs.

**Key questions**

**What is already known?**

- While there is a growing body of research on the design and function of global research partnerships that include or are based in low-income and middle-income countries (LMICs), less has been published about research consortia and networks.
- To our knowledge, this is the first work to identify the range of typologies and major design choices in developing health research consortia (RCs) and particularly for those addressing evidence gaps in LMICs.

**What are the new findings?**

- We found that across a wide range of RCs, the structure and function were determined by nine key design decisions that were explicitly or organically made in three domains: scope, organisational structure and funding decisions.
- RC models ranged from more structured functioning networks with large technical cores and strong central governing bodies to less structured models with minimal or no core and looser governing bodies, reflecting decisions made by organisers and influenced by a number contextual factors.

**What do the new findings imply?**

- There is not one ‘right’ approach to an RC, rather decisions are made to fulfil the values and priorities of the RC as a whole and can shift over time.

## Background

Forty years following the Alma Ata declaration,^1^ the world met in Astana in 2018 to reaffirm the commitment to primary healthcare (PHC) for all.^2^ Growing recognition of the central role of PHC to achieve universal healthcare and reach the Sustainable Development Goals has catalysed national and international commitments.^3–8^ However, the delivery of PHC in many low-income and middle-income countries (LMICs) is often weak, inequitable and of poor quality.^9^ Multiple stakeholders have recognised the need for better knowledge on how to measure and improve PHC to address these gaps and integrate relevant methodologies such as implementation and health policy research, identifying components of high-performing quality PHC systems and highlighting some of the areas where more knowledge is needed.^9–12^ One result of this work is a reflection of the need to better understand PHC knowledge gaps in LMICs and deploy resources most fit to uncover them.^13^ Research consortia (RCs) focused on health are well positioned to provide structure to accelerate research on identified knowledge gaps, often linking researchers across broad geographic distances or disciplines to focus on priority research agendas.^14–16^ These consortia can also provide opportunities to engage country-based stakeholders in prioritising and generating the research, creating collaborations to more effectively harness and use resources to address relevant questions, reduce research waste and enhance impact of empirical findings and build country capacity.^17–19^

While there is a growing body of research on the design and function of global research partnerships that include or are based in LMICs, less has been published about RCs. Research on what enables a productive collaboration found clear expectations of roles and emphasis on learning and innovation within defined parameters to be critical factors.^20–22^ Equity is an important factor in high-income country (HIC) and LMIC research partnerships, with member responsibility, effective leadership, capacity building and promotion of equity and inclusion vital in effective collaborations.^23 24^ Funders’ roles and responsibilities are also important in designing funding schemes to incentivise equity-oriented research in LMICs.^25 26^ Research on overall aims and structures of RCs has looked at factors including multiplicity of goals, number of partners, nature of their management and composition of partners.^24 27 28^ However, gaps have been identified in the understanding of how research networks operate including operational and structural decisions, especially in LMIC/HIC collaborations.^29^

In 2016, Ariadne Labs received funding to explore the potential for an RC designed to address prioritised gaps in evidence for measurement and improvement of PHC in LMICs.^13 30 31^ An initial step was an exploration of models for RCs. We describe the results of a landscape analysis and follow-on interviews to identify different typologies and structures of health-focused RCs, their format, operational approach and key design choices.

## Methods

### RC definition and eligibility

Drawing from existing work,^32 33^ we defined an RC as a community of individuals or organisations with shared interest that engages with one another to collaborate, share and develop resources in order to target and conduct more effective, efficient research. For consistency, we refer to these groups as RCs, but they may refer to themselves as consortia, communities of practice, initiatives, networks or platforms.

### Landscape analysis

We conducted an initial screen to identify relevant RCs through the three approaches: a Google search using combinations of the following terms: *health*, *global health*, *primary health care*, *research*, *consortium*, *network*, *hub*, *system*, *scientific advisory committee*, *steering group*, *collaboration*, *initiative*, *partnership* and *coordinating centre* and geographic terms in an effort to ensure the inclusion of RCs from the range of LMIC regions, using the World Bank classification of LMICs^34^; a search of the Encyclopedia of Associations, a directory of more than 135 000 associations, non-profits and societies worldwide^35^ using combinations of terms such as *global health research network* and *primary health care* *RC* (see online supplementary file 1 for full list of search terms); and from key informants including members of the technical advisory group convened for the PHC RC development work^13^ and snowball from key informant interviewees.

10.1136/bmjgh-2019-001450.supp1 Supplementary data

#### Initial inclusion criteria

For each separate search term, we considered the first three pages of results.^36^ For all searches, we looked at the results header and summary. An entity was included in the initial list if:

1. It contained relevant terms like “research network”, “consortium” or “initiative” in its title.
2. It had not already been included from a previous search or recommendation.

**Figure 1:**
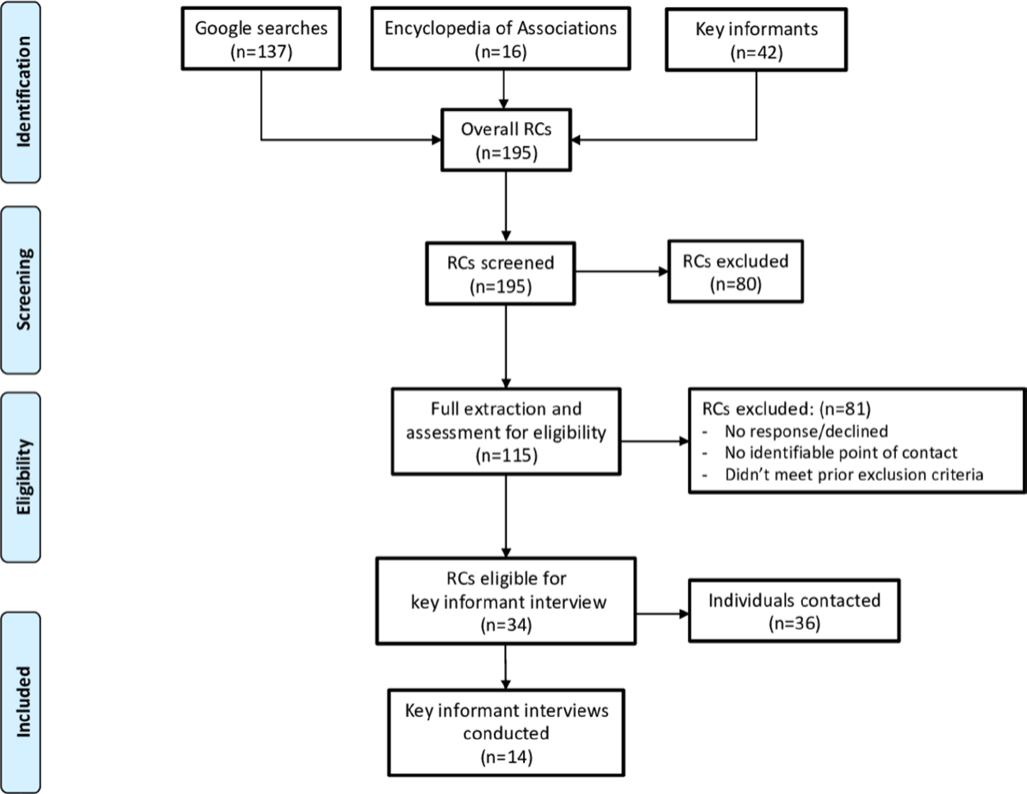
CONSORT diagram. CONSORT, Consolidated Standards of Reporting Trials; RCs, research consortia.

We did not exclude any RCs for location, the geographic focus of their research or health research agenda. Due to time and resource constraints, we restricted our search to English language resources. See figure 1 for a Consolidated Standards of Reporting Trials (CONSORT) diagram.

### Data extraction

We developed a standardised data extraction tool designed to capture details on the structure, funding, and outcomes of the RCs. This tool was used to extract organisational components such as the coordinating entity, funder, year established, size, composition of RC membership, whether its membership or research focus included LMICs, primary research area and main goals. The data extraction was made by AV and then checked by other authors for accuracy.

Entities were eligible for full extraction if the RC met the following criteria:

1. Met the definition of an RC as detailed above.
2. Health or healthcare focused.
3. Active in the last 10 years at time of review (since 2008).
4. RC description and relevant materials were in English.
5. Had an active website with contact information for leadership and description of participants.

### Key informant interviews

Two of the authors (AV and LRH) reviewed the list of potential RCs to contact and determined an order of priority for outreach. These included RCs that worked with or in LMICs or whose area of focus included PHC. We used RC websites to identify technical and administrative leads and funders. We contacted 36 individuals via email to request an in-depth interview.

We developed and piloted a semistructured interview guide that addressed the key themes of governance and administrative coordination, RC research agenda, topical focus, request for proposals (RFP) process, data management and dissemination of research. Interviewees were asked to describe their RC’s respective models and key decisions. We also asked interviewees to reflect on successes and lessons learnt from their respective RCs, and for recommendations on other RCs to consider in our landscape survey (see online supplementary file 2 for the interview guide). Interviews were conducted until thematic saturation was met (n=14).^37^ After obtaining verbal permission, interviews were audio recorded and transcribed verbatim for analysis. No identifying quotes were included, and audio recordings were destroyed after transcription.

10.1136/bmjgh-2019-001450.supp2 Supplementary data

### Analysis

We analysed key informant interviews using thematic content analysis,^37 38^ with a priori themes providing the basis of analysis focused on functions of the core, governance and decision making, funding sources, research agendas and focus areas. These results were combined with data extracted from RC websites to develop the groupings of major design choices and RC models and structures. Additional information on the contextual factors that influenced decisions on organisation, funding, membership and other key characteristics was also synthesised.

### Patient and public involvement

Patients were not involved in this research.

## Results

### RC components

We identified and reviewed 195 RCs (see online supplementary file 3 for full list). Of these, we excluded 80 after the initial screening, most commonly for not meeting the definition for an RC (see figure 1).

10.1136/bmjgh-2019-001450.supp3 Supplementary data

The remaining 115 RCs represented a range of geographies from a single country to multiple regions. They ranged in size from fewer than 10 to over 100 members (table 1). Funders were typically government agencies, large foundations or national research funders, such as the National Institutes of Health in the USA, or the Department for International Development in the UK. However, some were self-funded by their coordinating institution, and a few functioned as non-profit entities.

**Table 1:** Research consortium summary components

Indicator	Common characteristics (n=115)
Primary funder	Government agency: 37% (n=43)
	National research council: 22% (n=25)
	Self-funded: 14% (n=16)
	Foundation: 10% (n=12)
	Non-profit: 4% (n=5)
	Unknown: 12% (n=14)
Year established	Between 2013 and 2018: 26% (n=30)
	Between 2008 and 2012: 29% (n=33)
	Prior to 2008: 36% (n=41)
	Unknown: 9% (n=10)
Number of participants	>25 members: 46% (n=53)
	10–25 members: 27% (n=31)
	<10 members: 14% (n=16)
	Unknown: 13% (n=15)
Type of participants	Institutions: 47% (n=54)
	Individuals: 29% (n=33)
	Both: 20% (n=23)
	Unknown: 4% (n=5)
Geography of members and/or research focus	High-income countries only: 54% (n=62)
	Low-income and middle-income countries: 44% (n=51)
	Unknown: 2% (n=2)
Main topic	Health research: 17% (n=19)
	Primary healthcare: 14% (n=16)
	Other/specific cause: 14% (n=16)
	Healthcare, systems or policy: 12% (n=14)
	Infectious diseases: 10% (n=11)
	Population health: 10% (n=11)
	Mental health: 9% (n=10)
	Child health: 8% (n=9)
	Non-communicable diseases: 4% (n=5)
	Environment: 3% (n=4)

Member types varied, including research institutes, individual researchers or a combination of both. Member responsibilities also varied; some larger RCs allowed members to sign up without specific requirements or responsibilities, while smaller ones often had explicit expectations of a high level of involvement from each member.

While all surveyed RCs focused on health or healthcare research, there was a range of main topics of focus. The most commonly occurring topics included PHC (n=16), infectious diseases (n=11) and mental health (n=10) (table 1).

### Key informant interviews

We identified 34 RCs for key informant interviews based on geographic and subject area relevance. We sequentially contacted 36 administrative leads, technical leads and funders, and completed 14 interviews representing 13 RCs; eight individuals declined or failed to participate in the interview, and we did not receive a response from a further 14. RCs that included LMIC members had a higher interview rate (50%) compared with HIC-only consortia (30%). Six of the individuals interviewed described work with RCs that had HIC-only focus, and eight worked with RCs that had global or LMIC focus. table 1 shows funder, size, member composition, geographic focus and main topic of the eligible RCs, including those interviewed.

### Consortia designs and typologies

We found in practice RCs develop in an organic way, rather than aiming for a specific typology from a list of potential models. Instead the RCs were often defined by a series of key decisions around RC scope, administration, structure and funding, sometimes at the start and often adapted over time. As one RC administrative lead noted: ‘*The format kind of happened, rather than being chosen, [and] changed over time in terms of bringing about more structure*’. From RC text review and key informant interviews, we identified nine major design choices for how the RCs were organised, administered and funded to develop what were often relatively unique structures and functions (table 2).

**Table 2:** Definitions of nine major areas for research consortium (RC) design decisions

Major design element	Definition
1. Mission	The core purpose and focus of the RC.
2. Area of focus	An RC’s research agenda, the flexibility of the agenda and the geography considered in the RC’s research.
3. Core support	The functional support mechanisms for the RC.
4. Location of the core	Where—geographically and in what type of institution—the core is based and whether the core rotates.
5. Choice of leader	The specific individual(s) designated to hold the position of leader and whether the role is constant or rotates.
6. Governance	How decisions are made and how much power the leadership has to make decisions.
7. Membership	The composition, responsibility and eligibility of members in an RC.
8.Funding source for RC core operations	The entity or entities responsible for providing the funds used to operate the RC.
9.Funding sources and process for RC research	How funding for RC research is procured.

### Major design choices

We grouped the critical design decisions identified into three domains: I: scope; II: organisational structure and III: funding (table 3).

**Table 3:** Nine major areas for design decisions and examples of choices made by reviewed research consortia (RC)

Domain I: scope decisions		Domain II: organisational structure					Domain III: funding decisions	
1. Mission	2. Area of focus	3. Core support	4. Location of the core	5. Choice of leader	6. Governance	7. Membership	8. Funding source for RC core operations	9. Funding sources and process for RC research
Exclusive focus on research and developing knowledge base. ­ Research as well as researcher capacity building component.	Research agenda: predetermined, single population or clinical area. ­ Broad agenda across all RC. ­ Different research areas within the RC. Flexibility of agenda: fixed agenda. ­ New areas could be introduced. Geographic focus: fixed focus. ­ New focus could be introduced.	No or minimal core support. ­ Administrative core support, largely focused on communications, convening, network management and alerting members to funding opportunities. ­ Full technical support team, in addition to administrative support, fundraises, develops RFPs and provides statistical support.	Geographic location: core located in an LMIC. ­ Core located in an HIC. ­ Core location rotates. ­ Type of institutional home: core operates within a university research centre. ­ Core operates as a non-profit or for-profit entity.	Rotation: fixed role with one institution funded or designated to provide leadership (or leadership team). ­ Leader, or leadership team, changes over time. ­ Composition: led by a team. ­ Led a single individual. ­ No identified leader, full RC membership leads.	Full membership body determines research agenda and other key decisions. ­ Full membership body has some say, but final decision-making authority belongs to steering group. ­ A smaller steering group, comprising members and possibly funders, makes all decisions. ­ A smaller steering group makes some decisions, with final authority belonging to a single person.	Composition: individual institution/network. Both Responsibility: members join but no obligations. ­ Members apply for funding, opt into or out of proposed research projects. ­ Members fundraise and determine research agenda. ­ Different levels of membership, aligned with different expectations of responsibility. ­ Eligibility: members self-select in with no eligibility criteria. ­ Members self-select in with eligibility criteria (eg, LMIC and academic institution). ­ Members have to meet eligibility criteria and then apply, for example, respond to RFP. ­ Members are sought out and invited by RC leadership.	Single funder. ­ Multiple funders. ­ Self-funding: members pay dues or other membership fees. ­ Members’ research funding provides % to RC for core support.	Research funded by the RC core or single funder. ­ Grant writing by members, coordinated by the RC core. ­ RC core as a member of research grants. ­ Blended approach (eg, RC raise funds and members write grants with and through the RC).

HIC, high-income country; LMIC, low-income and middle-income country; RFP, request for proposal.

### Domain I: scope decisions

The major decisions related to scope included mission and area of focus, although decisions around duration and funding availability were also relevant.

#### 1. Mission

For some RCs, the mission was purely research and the development of a broader knowledge base for their respective field of study or studying interventions to improve relevant field outcomes. For other RCs, building research capacity was an explicit component of their mission as well.

Several RC leaders discussed their members’ desire for more career development or training opportunities. One administrative lead explained,

This was an administrative network that facilitated opportunities in PHC research and the sharing of PHC relevant resources and work amongst PHC related researchers and knowledge users – so the main goals were capacity building in PHC research and knowledge transfer.

While some RCs faced funding restrictions on such activities, others found this was critical to furthering the goals of their RC charter.

#### 2. Area of focus

The area of focus encompasses an RC’s research agenda, the flexibility of the agenda and geographic focus. Options for the research agenda included focusing on a predetermined and static population or clinical area; a broad agenda across the RC; or different, more specific research areas within the RC. The RCs identified ranged significantly in their geographic focus, from a single region to global research.

One large RC had an expansive agenda, with different research areas driven by different subgroups. The RC could support a broad agenda, and because members helped obtain funding for their respective research areas, it was within its capacity to do so. Smaller RCs tended to have more focused agendas such as HIV in specific geographic areas or mental health in a single country.

The RC could determine a fixed agenda at the outset of its work, or the agenda could be iterative, allowing new areas to be introduced through a decision-making process. One RC’s technical lead described a set overall research agenda but a flexible approach to specific foci over time. Geographic focus could also change. In one RC with research partners in HIC and LMIC countries, specific targeted regions or countries were determined in each funding cycle, as partners had to recompete each time.

### Domain II: organisational structure

We considered decisions around administration and operationalisation of an RC to include five design choices: core support, the location of the core, choice of leader, governance and membership. Leadership and governance are closely linked but we separated how decisions were made (governance) from the specific choice of individuals leading the RC.

#### 3. Core support

We defined core support as the scope and degree of support in the RC. These could include communications, convening, fundraising, grant management and statistical and analytical support of the research work of the consortium. We identified three main typologies of core support, including no or minimal core support, primarily administrative core and a full technical and administrative core support team.

With a minimal core, support might be provided in a part-time capacity and be typically purely administrative. This approach was seen in an environment where members have high technical and human resources capacity, an ability to proactively take on and share the operational work. One RC was composed of well-funded, technically adept member institutes and preferred a minimal core:

Projects and initiatives are led by member agencies, coordinated by [a part-time administrator], but those aren’t driven by the secretariat. Each [member] leads, and sometimes funds, necessary things for each project. *Administrative lead*

The second and most common typology of core support–a purely administrative core–typically involved a full-time staff providing administrative support with communications and convening efforts. This focused core was identified as important to demonstrating the value of the RC to potential or existing members.

We do a lot of monitoring of funding opportunities in the coordinating center. We spread the word to members who apply for funding, usually in partnership with other members. *Administrative lead*

A more comprehensive core can provide technical and administrative support including raising resources (fundraise, develop RFPs and grant writing) and scientific support (statistical or measurement support or other technical expertise). For a large network closely involved in driving the research output, providing strong technical support was an important way of demonstrating value to its members. One RC supported several dozen research protocols and established a core team capable of supporting a robust research infrastructure with multiple field sites and across multiple fields (informatics, ethics and laboratory).

#### 4. Location of the core

Decisions on the location of the core included geography and type of institutional home. RC core locations vary by permanence (fixed or rotating) and country income level. Potential institutional types included operating within a university research centre, non-profit or for-profit entity.

One RC that had existed for several years and included members from across the globe had a core permanently based at an HIC university. The original decision for the location was for convenience, according to the RC’s administrative lead, and while ‘*it makes sense to stay there now…we don’t have to stay. We take all opportunities to market we’re not (the HIC university) but a global network, don’t want to exclude people*’.

Another consortium tried to balance the question of equity and access through parallel offices in an HIC and an LMIC, while in another, the full core and hosting duties rotated between institutions at LMICs to build capacity and reduce the burden for any individual institution.

#### 5. Choice of leader

Design decisions around the choice of leader included fixed or rotating, individual or team (at a single institution or across multiple ones) and what functions the leadership had responsibility over. Leadership could be a fixed role with one institution or individual designated to provide leadership (or a leadership team) on an ongoing basis. Alternately, the leader or leadership team changed over time and rotated across institutions.

In one RC with HIC and LMIC members, the leadership team comprised a board of governors, each serving a 2–3 year term that could be renewed once. The goal was to found the RC with more prominent leaders who could help establish it, and then create opportunities for researchers from LMIC countries to participate in leadership through the life of the RC.

In another RC, the administrative leader was permanent, established as a separate entity from the members conducting the research, who had to reapply to the RC every funding cycle. The goal was to separate responsibilities and authority (researcher vs administrator).

#### 6. Governance

Decisions around governance included how decisions were made and how much the power rested with the leadership versus members. We identified four main options: the whole membership determines the research agenda and other key decisions; the whole membership, or representatives, has a say in decisions, but final authority belongs to a smaller steering group; a smaller steering group, comprising members and possibly funders, makes all decisions; or a smaller steering group, comprising members and possibly funders, makes some decisions, with final authority belonging to a single person. In some RCs, a substantial amount of activity took place through member-led working groups, with members encouraged to take direction on specific research themes.

In one RC, in which members were research institutes, each was represented in the governing body.

Each member has a person on the board… The board has the decision-making authority to say we want to do research geared toward [x or y area]. *Administrative lead*

That body was responsible for all decisions around the research agenda and operations, requiring a high level of consensus for any activity.

In another RC with research institutes as members, a representative steering committee was nominally in charge or setting priorities. In practice, however, a smaller executive committee held decision-making power:

We [the smaller executive committee] do the deep dive in terms of directions and questions, especially in terms of how best to allocate dollars in the grant. Then we propose to the steering committee which almost always agrees. *Technical lead*

#### 7. Membership

Membership design decisions included composition, responsibility and eligibility. For composition, RC members included individuals, institutions or other groups such as networks, or a combination of individuals and institutions. Some RCs were strict, for example, only allowing individual researchers or clinical practices, while others allowed more flexible participation. Responsibilities of members ranged from no obligations to varying expectations around membership fees, responsibility for funding application, engagement in defining the research agenda and playing a supportive role in RC management.

In one RC, the technical lead explained, people ‘*basically just have to say they’re interested and they can join as a “mailing list member”,’’* whereas *‘“consortium members”* *take on a topic area and agree, formally, to serve on the executive committee’*. These tiers developed as the RC’s governing body identified member capabilities and interest.

### Extent of formal organisational structure

We also identified three levels of organisational structure based on decisions on governance, membership expectations and core support. The most structured RCs typically maintained a strong central governing body, large technical core and high membership responsibility. The RCs in this model tended to be funded by national research funders or private foundations. Membership size was typically 10–25, with members comprising institutions including hospitals, research institutes and universities.

RCs with intermediate levels of structure had a wider range of models but usually had a governing body with varying degrees of centralisation, a core that could be purely administrative or provide some technical capacity and moderate membership criteria with varying levels of responsibility. Across the RCs, this model was usually funded by universities, national research funders or foundations. Membership size ranged dramatically, with individuals or institutions as members.

The least structured RCs were often led by representatives from each membership organisation, with a minimal core, and low mandatory membership responsibility. Members drove the research agenda and their own research priorities, collaborating where they found it appropriate. RCs that fit this model were commonly funded internally or by diverse stakeholders. Membership size was typically more than 25 with member composition including researchers, clinicians, policy makers and research institutes.

However, we found that organisational structure was often fluid. For example, after a few years of existence some found that initial structures were not achieving their goals, and adaptations were made.

### Domain III: funding decisions

Decisions on the funding source for the RC core as well as the funding process for research were important to the design and structure of RCs.

#### 8. Funding source for core operations

Funding decision were both for the core and for research. Sources for each could include funding by a single funder; multiple funders; self-funding, with membership fees sufficient for the RC structure and functions; and a system in which members’ research funding includes a percentage set aside for core support.

An RC with operational funding from multiple sources did this as a requirement by the original funder:

They committed to giving [a certain amount] over 5 years and required [a match] over 5 years from others. *Administrative lead*

Often the goal in a case like this would be a more sustainable funding base, so if one source ceased to provide funding, the RC could continue its work.

One RC whose members were research institutes provided its own operational funding. The administrative lead noted, ‘*we are a membership organization. Each member pays dues… [and* *] the operating budget comes 100% from member dues*’*.* While self-funded models were uncommon, this RC had a sufficiently large and well-resourced membership base to facilitate this approach.

Another RC was becoming self-sufficient. According to the technical lead, ‘*[we have] one facility fee that applies to all faculty… we put on grants that goes to support some of the infrastructure in [the LMIC partner country].* This approach resulted from a strong collaboration of the members’ research projects and the RC’s technical support.

#### 9. Funding sources and process for RC research

Options for funding sources and process for RC research included a range of approaches: fundraising solely the responsibility of the RC core or single funder; grant writing by members coordinated by the RC core; the RC core as a member of research grants (eg, providing statistical support); or a blended approach.

At one RC, the technical core coordinated with members to develop research proposals, with a strict consultation and review process for grants developed under the RC’s auspices. The technical lead explained it was logical for the core to support this work, as ‘*they need the infrastructure to do their research, and have successful competitive research work going on*’.

Another RC had a blended approach, with funds at the core’s disposal specifically to fund research, for which it set up competitive applications. Additionally, the RC served as a member of research grants:

We have two types of funding: some is flexible, our core funding comes from this. It’s entirely up to us how to spend it… we get the approval of the working group… they ensure quality and technical rigor, if we want to issue a call, people apply, there is an independent review process. The rest is specific, work from funders [on specific projects]. *Technical lead*

### Equity in HIC and LMIC research partnerships

While the nine decision areas were important for RCs regardless of membership and areas of focus, RCs that included HIC and LMIC members raised specific considerations. Leaders expressed that issues of power in a number of areas were common in these partnerships and needed to be directly addressed, and these influenced some of the nine design choices.

Ensuring all member voices were heard in RCs reaching across geographies and member backgrounds was an important priority. Sometimes this meant adapting communications technology to be more inclusive, such as using smart phone apps instead of email, and other times this meant rotating annual meeting locations to include the full array of member countries. This is the case regardless of membership but made more important when a power imbalance exists.

For me the biggest thing with running a network is really good communication with everyone. Making sure they’re received, understood, [and that] messages go all the way out to the field level. *Administrative lead*

Leaders also highlighted the importance of equity in treatment of all members regardless of background, as greater importance in contexts of greatly differing access to resources and opportunities. Some RCs with partners across HICs and LMICs created formal leadership sharing models, in which every project had an HIC and an LMIC partner. Intentionally rotating meeting locations and the geography of the leadership were also important steps to balancing ownership considerations. Said one technical lead, ‘*by decentralizing the project across countries, rotating the annual meeting place – it creates much more ownership of what’s going on*’.

## Discussion

We found in this landscape analysis that the structure and function of RCs were determined by decisions made in three domains: scope, including the mission and area of focus; organisational structure, including role and location of the core entity, leadership, governance and membership eligibility and responsibility; and funding decisions, including the funding source for RC operations and the funding sources and process for RC research. We found no two RCs were identical, with differences in design in one or more of these areas often based on their goals, needs and resources (see Box 1).

Box 1 **The key decisions through the lens of an existing research consortium (RC)**

The AMPATH Research Network was established in 1998 and operates as a research partnership codirected by investigators in the USA and in Kenya.^41^ It maintains a broad research focus, with working groups conducting research projects in primary care, paediatrics, reproductive health and basic science, among other areas.^42^ This is an example of an intermediately structured RC. It has a large technical core with governance balanced between a small leadership team and the participation of the larger body of members.^41–43^ As with many of the RCs we reviewed, some aspects to this RC were fairly common among RCs, while other design decisions were less typical, made in response to the RC’s specific needs.

- Mission: the mission focuses on capacity building in addition to research, with strategic priorities that include expanding the population of trained researchers. 41
- Area of focus: the RC originally focused on HIV/AIDS research but has expanded over time. It is structured to support this expansion, with different research areas currently harnessed into 10 subgroups.^42^ The geographic focus on low-income and middle-income countries (LMICs) has not changed.^41^
- Core support: this RC is supported by a large technical and administrative core that provides assistance with proposal development, communication, biostatistics and data management, and laboratory support.^42 43^
- Core location: the RC core is large and has components located both in high-income countries and LMIC countries.^41 42^
- Choice of leader: leadership is permanently located, with coleads based in the USA and in Kenya.^41^
- Governance: the RC governance is driven by a smaller steering group with final authority belonging to the codirectors.^41 42^ However, the programme structure includes 10 research working groups that are responsible for much of the development of research studies.^41 42^
- Membership: membership is comprised of institutions, and there are strict membership criteria with high responsibility and expectations of participation from members.^44^
- Funding source for RC core operations: the funding source is composed of diverse funders, including government agencies, private foundations and a percentage of members’ research funding that helps to fund the RC core’s research support services.^43^
- Funding sources and process for RC research: the RC actively coordinates grant writing with members and may serve as a member of research grants through the use of its research support infrastructure.^41 43^

We did identify some basic *structures* that seem universal across all RC types: designated leadership, administrative function and membership. What drives the design of these structures was influenced by a number of factors including the desired role of these different players, the funding landscape and level of funder involvement in decision making. Core structures were typically administrative, and located in an HIC, often due to availability of resources and funders. While formal tools were not typically used a priori by RCs for design or decision making, a few adopted more structured consideration processes to modify their design as time went on.

The extent of formal structure in the RCs reviewed ranged from more structured functioning networks with large technical cores and strong central governing bodies to less structured models with minimal or no core and looser governing bodies. Factors shaping some of these decisions included location of the core, funding source and mission. RCs with a mission that included capacity building, for example, might need a larger technical core in order to provide more junior research partners with additional support in developing research proposals and conducting data analysis.

In contrast, we saw the most variability in decisions around *membership* expectations and responsibility. This was influenced by field, by the goal of the RC, for example, prioritising accessibility to the broadest possible audience or to a specific subset of people, by the expectations of the governing body and by the scope of the RC. Highly variable approaches were also taken to decisions around funding sources and flexibility of funding. The RC’s research focus and scope, its members’ role in fundraising and its leaders’ connections and proactivity in fundraising efforts were major contributors to the availability and sustainability of funding sources for RC operations and research.

We identified key contextual factors such as existing resources and donor involvement that influenced design decisions throughout the RC lifecycle. We did not find a single best-practice approach to governance and decision making; rather, they typically depended on the RC goals and organisation. In some RCs, the driving priority was to be as democratic as possible, while in others, members looked to a small steering group to shape the research agenda and make daily decisions. This range in experience is consistent with the findings of Fair and colleagues,^39^ who showed collaborations tended to range from more formal and bureaucratic to more informal and participatory, depending on the needs of the group. *Research priorities* and scope were typically determined either by an RC’s highest decision-making body, which could include funders or the entire RC membership. Funders could have a role in shaping governance structures too, if they played a founding role in the RC, but otherwise the approach appeared determined by the ambitions of the group and the priorities of its founders.

The relationship that develops between an RC’s members, leadership and coordinating bodies is important to how the structure forms and changes shape and is considered one of the more intangible outputs of the research partnership.^14 28^ For example, de-Graft Aikins and colleagues^14^ found that building trust, respect and openness between members of the core working group can shape RC outputs and responses to research priorities; this could happen organically as an RC thrives and endures (or does not), or be taken into consideration intentionally by an RC taking stock of its priorities and focus with an eye towards the future.

In their work on successful research collaborations, Parker and Kingori^23^ demonstrated that promoting equity in an environment of compromise was particularly important for research in lower income settings where existing resource imbalances could be exacerbated if not handled proactively. For example, planning and resourcing mentorship between HIC and LMIC research partners has been considered critical to ‘leveling the playing field’ in research,^15^ activities some RCs were able to achieve in their scope. Pratt *et al* 24 suggested that inclusion involved considerations of both *who* was invited to be present as well as *how* those invited were involved recognition of which seemed to be associated with some RCs’ efforts to reduce barriers for LMIC researchers to join through looser membership criteria. The need to promote equity and inclusion are important considerations when making RC design decisions.

### Limitations

This research included several important limitations. The landscape review was limited to English language search, causing important linguistic and regional limitations. The search process was thorough but not exhaustive; it is possible that not every potential RC that fit our definition was included if, for example, it used a name that would not have been picked up in our search or did not have an internet presence; furthermore, use of electronic publication databases could have uncovered RCs that did not appear in our search. We could not validate the information from the website or key informants, so some details may not have been fully accurate. We may have excluded some key informants based on website information. We were not able to necessarily capture the change over time in an RC if the key informant was new or the website did not detail changes over time.

## Conclusion

We found that rather than a discrete set of typologies, RCs are characterised by a common set of decisions made in three main domains that cover nine specific areas. The experiences of the RCs we spoke to suggested that there was not necessarily one ‘right’ approach. Decisions were made in an effort to fulfil the shared values, ethos and priorities of the whole RC and could shift over time.

This mapping of the key considerations and decision points on the development of an RC has been used in reaching consensus on the structure of a proposed model for a new PHC RC designed to address evidence gaps identified by LMICs to generate the knowledge needed by countries and implementers to continue efforts to strengthen PHC policies and delivery. The key decisions were used as a framework to guide a structured discussion, using nominal group technique,^40^ for the development of a new RC focused on PHC research in LMICs. It was found to be effective in reaching consensus in many of the three domains. Because RCs are becoming an increasingly common approach for LMIC-partnered and HIC-partnered research, this landscape analysis will be a valuable resource to health researchers striving to develop the research communities and needed infrastructure to improve health research globally.
