# Supplementary material for: It takes a community: a landscape analysis of global health research consortia
Source: BMJ Glob Health. 2019 Aug 16;4(Suppl 8):e001450. doi: 10.1136/bmjgh-2019-001450 (PMC6703292; doi:10.1136/bmjgh-2019-001450)
Supplement: Supplementary data [file bmjgh-2019-001450supp001.pdf]

Supplementary File 1. Key words for online search

<u>Google search</u>:
Health research network
Global health research network
Primary health care research network
Health research consortium
Global health research consortium
Primary health care research consortium
Health research coordinating center
Global health research coordinating center
Primary health care research coordinating center
Health research hub
Global health research hub
Primary health care research hub
Health research system
Global health research system
Primary health care research system
Health research scientific advisory committee
Global health research scientific advisory committee
Primary health care research scientific advisory committee
Health research steering group
Global health research steering group
Primary health care research steering group
Health research collaboration
Global health research collaboration
Primary health care research collaboration
Health research initiative
Global health research initiative
Primary health care research initiative
Health research network initiative
Global health research network initiative
Primary health care research network initiative
Health research partnership
Global health research partnership
Primary health care research partnership

<u>Targeted region Google search</u>:
Using term “health research consortium” and “health research network” (two of the most productive terms initially), we added in a targeted geographic arm to obtain fuller representation of LMIC World Bank regions.
[Central Europe OR Eastern Europe] AND health research network
[Central Europe OR Eastern Europe] AND health research consortium
[Central Europe OR Eastern Europe] AND primary health care research network
[East Asia OR Pacific] AND health research network
[East Asia OR Pacific] AND primary health care research network
[East Asia OR Pacific] AND health research consortium
[Latin America OR Caribbean] AND health research network

[Latin America OR Caribbean] AND health research consortium
[Middle East OR North Africa] AND health research network
[Middle East OR North Africa] AND health research consortium
[Middle East OR North Africa] AND primary health care research network
South Asia AND health research network
South Asia AND health research consortium

Encyclopedia of Associations:
Global health research network
Primary health care research network
Global health research consortium
Primary health care research consortium
Global health research coordinating center
Primary health care research coordinating center
Global health research hub
Primary health care research hub
Global health research collaboration
Primary health care research collaboration
Global health research initiative
Primary health care research initiative
Global health research network initiative
Primary health care research network initiative
Global health research partnership
Primary health care research partnership
