# Supplementary material for: It takes a community: a landscape analysis of global health research consortia
Source: BMJ Glob Health. 2019 Aug 16;4(Suppl 8):e001450. doi: 10.1136/bmjgh-2019-001450 (PMC6703292; doi:10.1136/bmjgh-2019-001450)
Supplement: Supplementary data [file bmjgh-2019-001450supp002.pdf]

Supplementary File 2. Key informant interview guide

Key Informant Interview guide

**Summary information**	
Research network:	
Organization:	
Name:	
Title:	
Contact info:	
Main role:	
Date started with the research network:	
Date of interview:	

**Overview of Research Network (RN)**		
A. Makeup and key components of research network (goal to fill in gaps and/or confirm details of the entity)	A4. What year was the network established?	
	A5. How many members are in the network?	
	A7a. What geographies do members include?	
	A7b. What geographies are included in the research focus?	
	A9. What were the original goals of the network?	
	A10. Can you describe the overall structure, and format, of the network?<br>*Stable group, change over time, multiple initiatives under one group etc.*<br>*Format - models might include hub and spoke, democracy, classic trial network, annual membership*	
	A11. What is the timeline of the consortium? *(Goal to exist permanently, pre-set length, as funding allows?)*	

**Structural Information**		
Note: When we talk about *coordination* and a *coordinator*, as below, we mean some sort of central entity, such as a core group, secretariat or administrator, responsible both for guiding the network, its strategic planning, and decisions, as well as the administrative functions of maintaining communications, budgets, and other core operational functions.		
B. Governance and Leadership	B1. How is the network coordinated? *How was this entity or format chosen? Has this changed over time?*	
	B2. What is the budget for coordination?<br>*What % is used for administration? For coordination? If RFP, what % used during RFP and afterwards? What % budgeted for FTE?*	

1

	B3. Who provides funding to the consortium as a whole?<br>*How are research funds identified? Who controls the funds (both for consortium and for research), and how are they distributed?*	
	B4. What are the main goals of the consortium?<br>*How were they developed? How have they changed over time? What drove those changes?*	
	B5. What determines eligibility of a researcher or organization to participate?<br>*Responds to an RFP? Membership? Asked to participate? Other?*	
	B6. What are some administrative challenges that the coordinating entity has faced? How have they evolved or been resolved?	
C. Research agenda	C1. Who sets the broad research agenda? What are the broad themes?	
	C2. How are research questions identified and prioritized?<br>*Centrally, democratically?*<br>*How has this changed over time? Describe the process.*	
	C3. When there is conflict about prioritization of general areas, how is it resolved?	
	C4. Does the network model help, or hinder, the process of identifying and prioritizing the agenda?	
D. Focus areas	D1. How are specific research topics chosen? How are researchers chosen?<br>*Can any member/participating organization propose topics?*	
	D2. If competitive, who designs the RFP? Who reviews?	
	D3. How many grants or projects are administered in a year?<br>*What is the target amount? How has this evolved over the course of the entity?*	
E. Data Management	E1. Is cross-site/project data collection a core function?	
	E2. Are data from projects shared across research groups?	
	E3. What are the protocols and policies for data sharing and utilization?	
	E4. Who has access to data?	
F. Dissemination of consortium findings	F1. What is the dissemination strategy?<br>*E.g. individual responsibility of researchers, role of coordinating groups funding, central support*	
**Overview and Future Directions**		

2

Note: The ultimate goals of the consortium are to (i) develop a robust Primary Health Care (PHC) global research network structure, and (ii) to secure the resources needed to carry out prioritized and policy-relevant PHC research to support better measurement, implementation, and improvement efforts in priority areas for PHC in lower and middle income countries. The research consortium's work is designed to support country and global efforts to build the high-quality PHC systems that are needed to reach effective universal health coverage and the health-related sustainable development goals.		
G. Overall strengths, weaknesses, opportunities, challenges, etc.	G1. Knowing what you do now about the strengths and challenges of your network and its model, could you identify one or two critical areas that you would change? Why, and how?	
	G2. Likewise, please identify one or two critical areas that your network is doing really well with. Why do you think this is the case?	
	G3. Can you identify two or three factors of critical importance with respect to collaborations involving members from both high- and low-income countries?	
	Can you provide recommendations for other research networks and/or contacts that you think might be useful to our efforts?	
