# Supplementary material for: It takes a community: a landscape analysis of global health research consortia
Source: BMJ Glob Health. 2019 Aug 16;4(Suppl 8):e001450. doi: 10.1136/bmjgh-2019-001450 (PMC6703292; doi:10.1136/bmjgh-2019-001450)
Supplement: Supplementary data [file bmjgh-2019-001450supp003.pdf]

Supplementary File 3. Full list of 195 RCs reviewed (listed alphabetically)

1. A Research Collaborative for Global Health Equity
2. ABCD National Research Partnership
3. AcademyHealth
4. Africa Evidence Network
5. African American Collaborative Obesity Research Network (AACORN)
6. African Economic Research Consortium
7. African Health Initiative
8. African Rural Social Sciences Research Networks
9. Afrihealth Optonet Association
10. AIDS Clinical Trials Group
11. Alabama Practice Based Research Network
12. AMARI: African Mental Health Research Initiative
13. American Academy of Family Physicians National Research Network
14. Anti-Microbial Resistance Research and Development Collaboration Hub
15. Applying Science to Strengthen and Improve Systems (USAID ASSIST)
16. BC Primary Health Care Research Network
17. Campbell Collaboration
18. Canadian Coalition for Global Health Research
19. Canadian Primary Health Care Research and Innovation Network
20. CDC’s Healthy Brain Research Network
21. Central and Eastern European Research Network
22. Childhood Arthritis and Rheumatology Research Alliance (CARRA)
23. Cincinnati Lifespan Practice Research Consortium
24. Clinical Trials Central & Eastern Europe
25. Clinical Trials Network (CTN)
26. Collaborative Pediatric Critical Care Research Network
27. Consortium for Families and Health Research
28. Consortium for Oral Health Research and Informatics (COHRI)
29. Consortium of Universities for Global Health
30. COST Actions
31. Council on Health Research for Development (COHRED)
32. Cystinosis Research Network (CRN)
33. Defense Health Research Consortium
34. Design & Health Research Consortium
35. DREAMS Partnership
36. Duke Kunshan University Global Health Research Center
37. Duke Primary Care Research Consortium
38. Ebola Clinical Research Consortium
39. Ensuring Value in Research Funders' Collaboration and Development Forum
40. Epilepsy Foundation
41. EQUATOR Network (Enhancing the Quality and Transparency of Health Research)
42. ESSENCE on Health Research
43. EU-LAC Health
44. European Forum for Primary Care
45. European General Practice Research Network (EGPRN)
46. European Research Council

47. European Research Network for Out-of-Hours Primary Health Care
48. Evidence-Based Research Network
49. FDA Sentinel Initiative
50. German network on health equity
51. GESI, the Global Evidence Synthesis Initiative
52. Global Alcohol Policy Alliance
53. Global Alliance for Chronic Diseases (GACD)
54. Global and Territorial Health Research Network
55. Global Environmental and Occupational Health (GEOHealth) Program
56. Global Health Policy Research Network at the Center for Global Development
57. Global Mental Health Research Consortium and Scholars Program at Columbia University
58. Global Network for Women's and Children's Health Research
59. Global Network of World Health Organization Collaborating Centres (WHOCCs) for Nursing and Midwifery
60. Global Research Collaboration for Infectious Disease Preparedness
61. Guidelines International Network
62. H3Africa Consortium
63. Health Care Systems Research Network
64. Health Systems Global
65. Healthy Flint Research Coordinating Center
66. HMO Research Network
67. IMPAACT Network
68. INA-RESPOND: Indonesia Research Partnership on Infectious Disease
69. INDEPTH Network
70. Indiana Consortium for Mental Health Services Research
71. Infectious Diseases Research Collaboration
72. Institute of Cultural Affairs Middle East and North Africa (ICA-MENA)
73. Institute of Cultural Affairs Zimbabwe (ICA)
74. Intermountain Consortium for Child Health Services Research
75. International Collaboration for Participatory Health Research
76. International Network for Cancer Treatment and Research (INCTR)
77. International Phenome Center Network: A Harmonized Research Consortium for Innovative Global Health
78. International Severe Acute Respiratory and Emerging Infection Consortium, ISARIC
79. International Society for Pharmacoeconomics and Outcomes (ISPOR) Arabic Network
80. Irish Health Research Forum
81. ISPOR Latin America Consortium
82. Johns Hopkins Primary Care Consortium
83. Kundalini Research Network (KRN)
84. Latino Health Research Initiative
85. Long-Term Assistance and Services for Research (LASER)
86. Lymphatic Education and Research Network
87. Maternal and Child Health Research Consortium
88. McGill Primary Health Care Research Network
89. MedStar Research Networks
90. MENA Research Network
91. Mental Health Research Network
92. Midwest Area Research Consortium for Health

93. Milwaukie Global Health Consortium
94. National Child Abuse Defense and Resource Center (NCADRC)
95. Native Research Network
96. Neonatal Research Network
97. Network of AIDS Researchers of Eastern and Southern Africa
98. Network of Cardiovascular Disease
99. NIH Collaboratory
100. NIHR Clinical Research Network
101. NIHR Collaborations for Leadership in Applied Health Research and Care (CLAHRCs)
102. North American Primary Care Research Group
103. Northern Pacific Global Health Research Fellows Training Consortium
104. One Health Network South East Asia
105. Oxford-India Health Research Network
106. Patient Centered Outcomes Research Institute (PCORI)
107. Pediatric Neurotransmitter Disease Association (PND)
108. Physical Activity Policy Research Network
109. Physicians' Research Network (PRN)
110. Platform for Social Research on Mental Health in Latin America (PLASMA)
111. Population-based Research to Optimize the Screening Process (PROSPR)
112. Population Health Metrics Research Consortium Project
113. PR at McGill (PRAM)
114. Practice-Based Research Networks Resource Center
115. PREPARE, Platform for European Preparedness Against (Re-)emerging Epidemics
116. Primary Care Research Network
117. PRIMASYS
118. Professional Association of Health Care Office Management, Greater Phoenix Chapter
119. Public Health Research Consortium
120. PURE Network
121. Qualitative Health Research Network
122. Rare Diseases Clinical Research Network
123. RCMI Translational Research Network
124. Regional Network for Mental Health Research in Latin America (RedeAmerica)
125. Research Consortium: Novartis
126. Research Network on Youth Mental Health Care
127. Resiliencia
128. Resource Centre for Primary Health Care, ENHR Nepal Network
129. Road Traffic Injuries Research Network
130. Ryan White Special Projects of National Significance (SPNS)
131. Sax Institute
132. Scottish Oral Health Research Collaboration
133. Service Delivery Indicators (SDI)
134. Shriver National Institute of Child Health and Human Development (NICHD)
135. South Asian Hub for Advocacy, Research & Education on Mental Health (SHARE)
136. South East Asia Infectious Disease Clinical Research Network
137. South East Asia Research Network
138. South-Asia Research Network
139. Southern African Research Consortium for Mental health INTegration (S-MhINT)
140. Sub-Saharan African Network For TB/HIV Research Excellence

141. Sutter Health Research Network
142. System Integration and Innovation Research Network
143. The AMPATH Research Network
144. The Canadian Epigenetics, Environment and Health Research Consortium
145. The Cancer Research Network
146. The CAPS Health Sector Research Consortium
147. The Caribbean Consortium for Research in Environmental and Occupational Health
148. The Child Health and Mortality Prevention Surveillance (CHAMPS) Network
149. The Children's Interstitial Lung Disease Research Network (chILDRN)
150. The Cohorts for Heart and Aging Research in Genomic Epidemiology (CHARGE) Consortium
151. The Dartmouth Global Health Initiative
152. The Effective Health Care Research Consortium
153. The European & Developing Countries Clinical Trials Partnership (EDCTP)
154. The European Health Management Association (EHMA)
155. The German Network for Participatory Health Research
156. The Global Health Network
157. The Global Surgical Outcomes Collaboration
158. The Head Start Mental Health Research Consortium
159. The Health Care Research Collaborative
160. The Health Improvement Network
161. The International Epidemiologic Databases to Evaluate AIDS (IeDEA) Research Consortium
162. The Johns Hopkins Primary Care Policy Center
163. The MAPP Research Network
164. The Medication Management Research Network
165. The National Patient-Centered Clinical Research Network
166. The Ndlovu Research Consortium
167. The Norwegian Primary Care Research Network
168. The Occupational Health Research Consortium in Aviation
169. The OneFlorida Clinical Research Consortium
170. The Partnership for Global Health Research Training Program
171. The Pediatric Research Consortium
172. The Prevention Research Collaboration (PRC)
173. The Rebuild Consortium
174. The Social Determinants of Health Network
175. The United Kingdom Child Health Research Collaboration (UKCHRC)
176. The University of Toronto Practice-Based Research Network
177. The Urban Health Network for Latin America and the Caribbean (LAC-Urban Health)
178. The Washington University Pediatric & Adolescent Ambulatory Research Consortium
179. TMJ Association
180. Trans-America Consortium of the Health Care Systems Research Network
181. Translating Research into Action (TRAction) Project
182. Triangle Global Health Consortium
183. Type 1 Diabetes (T1D) Clinical Research Network
184. U.S. Department of Health and Human Services - National Institutes of Health - Eunice Kennedy
185. UCL Central and Eastern European Health Research Group
186. UK Prevention Research Partnership Consortium
187. United States-Latin America Cancer Research Network (US-LA CRN)
188. University of Chicago global clinical research network

189. VA Women’s Health Research Network
190. VECD Consortium; Vanderbilt, Emory, Cornell, Duke
191. Wellcome Trust
192. West and Central African Research and Education Network (WACREN)
193. WHO Alliance for Health Policy and Systems Research
194. Workplace Health Research Network
195. WWAMI region Practice and Research Network (WPRN)
